# Surfacing and diving behavior associated with thermal physiology in oceanic habitats of skipjack tuna (*Katsuwonus pelamis*) in the western north Pacific Ocean

**DOI:** 10.3389/fphys.2025.1462940

**Published:** 2025-01-31

**Authors:** Yoshinori Aoki, Takashi Kitagawa, Hidetada Kiyofuji

**Affiliations:** ^1^ Fisheries Resources Institute, Japan Fisheries Research and Education Agency, Yokohama, Kanagawa, Japan; ^2^ Graduate School of Frontier Sciences, The University of Tokyo, Kashiwa, Chiba, Japan

**Keywords:** skipjack tuna, vertical behavior, archival tag, migration, thermal physiology, thermoregulation

## Abstract

**Introduction:**

Thermal physiology is a pivotal biotic factor for the ecophysiology of commercially valuable tuna, influencing not only horizontal but also vertical behaviors. We aimed to examine how the thermal physiology of skipjack tuna (*Katsuwonus pelamis*, SKJ) can explain the differences in their vertical behavior, focusing on surfacing and diving, among various thermal environments during their northward migration in the western North Pacific.

**Methods:**

We analyzed archival tag data collected during 2012–2015, with individual time series (Fork length: 38–49 cm, N = 38) of swimming depth, water temperature, and peritoneal body temperature during northward migration from subtropical areas to temperate regions around Japan. We quantified surfacing and diving behavior as an index of vertical behavior and estimated the whole-body heat transfer coefficient (*λ*) during the cooling and warming phases associated with diving using body and water temperature records as indicators of thermal physiology.

**Results:**

In the southern mixed layer areas, SKJ were widely distributed at a depth layer <200 m, whereas they were restricted to the surface in the strong thermocline areas in the north. The dive duration was significantly shortened with a strong thermal gradient during northward migration. We observed minor to no differences in *λ* values between the cooling and warming phases in the southern areas, whereas the *λ* values in temperate areas differed by a factor of 2–3 between the phases.

**Discussion:**

Our findings of changes in *λ* values between the cooling and warming phases represent the first evidence of thermoregulation in SKJ. Surfacing preference behavior and short dive duration in temperate areas may be an avoidance of prolonged exposure to cold temperatures, a behavior commonly exhibited in other tuna. Moreover, we discussed how the changes in vertical behavior driven by thermal physiology can explain spatial heterogeneity in SKJ fishery grounds in the western Pacific Ocean.

## 1 Introduction

A core challenge in the ecophysiology of commercially valuable tuna is predicting how species respond to environmental changes, such as global warming, which is essential for interpreting their distribution, population dynamics, fishing strategies, and effective management (Lehodey et al., 2013; Dueri et al., 2014; Bernal et al., 2017; Bell et al., 2021). In particular, the vertical behavior of tuna is crucial for fishing activities, particularly regarding the overlap in depths and time between target species and gear settings by humans (Post et al., 2008; Lennox et al., 2017; Wright et al., 2021; Matsubara et al., 2024). Surface-oriented behavior renders tuna more vulnerable to surface fisheries than the behavior that keeps them at deep depth for a long time (Matsubara et al., 2024). Therefore, changes in vertical behavior across areas can be a good indicator to explain the spatial heterogeneity of fishery grounds (Forget et al., 2015; Scutt Phillips et al., 2017).

In 2022, Skipjack tuna (*Katsuwonus pelamis*, SKJ) was the third most exploited fish species globally (2.8 million tons), for the 11th consecutive year (FAO, 2022). SKJ catch in the Western and Central Pacific Ocean (WCPO) is predominantly exploited by purse seine (84% of 1,735,500 mt for 2022) and pole-and-line fisheries (8%), along with various “artisanal” gears (8%, Williams, 2023). These fisheries have been mainly operated in tropical and temperate areas (Williams, 2023) and rarely operated in subtropical areas, despite SKJ being widely distributed from tropical to temperate areas (Matsumoto et al., 1984; Wild and Hampton, 1994). Beyond fishery-dependent information, advancements in biologging technologies have improved the study of SKJ migration ecology in the WCPO (Aoki et al., 2017; Kiyofuji et al., 2019). SKJ exhibit long-distance north-southward migration, moving from subtropical areas to foraging temperate foraging grounds off Japan. Throughout the northward migration, the vertical distribution becomes shallower as latitude increases, likely due to the avoidance of lower temperatures (Kiyofuji et al., 2019). These field observations can explain the spatial gap in fishery grounds by improving our knowledge of the depths at which SKJ is distributed during migration. However, we still lack a clear understanding of how and why their depth changes. To address this gap, behavioral differences among thermal habitats should be evaluated in conjunction with the underlying physiological mechanisms.

Inferring thermal physiology is a key field for addressing the underlying reasons behind tuna behavior, as their unexceptionally elevated body temperature has ecological advantages in the expansion of thermal niches (Brill, 1994; Bernal et al., 2017). A key feature contributing to the ability to elevate body temperature above that of ambient water is the axial positioning of the locomotor red muscle, which reduces conductive heat loss to the environment at the body surface (Dickson, 1996; Graham and Dickson, 2000; Korsmeyer and Dewar, 2001). Furthermore, heat generated in the red muscle is effectively conserved by a complex vascular network of counter-current heat exchangers (retia mirabilia), which play a role in minimizing convective heat loss to the gills (Carey and Teal, 1966; Brill, 1994). Owing to their unique anatomy, body temperature during dives can be maintained relatively stable in tuna, a phenomenon known as short-term thermoregulation (Holland et al., 1992; Aoki et al., 2020; Hino et al., 2021). For example, Bigeye tuna (*Thunnus obesus*) inhabiting cool water below the thermocline quickly ascend to warmer surface water to rapidly absorb heat via conduction, as their body temperature approaches the ambient temperature (Holland et al., 1992; Boye et al., 2009; Hino et al., 2021). Subsequently, they immediately descend back below the thermocline while suppressing the decrease in their body temperature. This remarkable thermoregulation is a result of the physiological modulation of thermal conductance during the cooling and warming phases associated with their vertical movements (Evans et al., 2008; Holland et al., 1992; Malte et al., 2007). The degree of physiological modulation, often expressed as the whole-body heat transfer coefficient (*λ*), exhibits variability across tuna species (Aoki et al., 2020; Kitagawa et al., 2006; Kitagawa et al., 2022). Regarding thermoregulation in SKJ, only laboratory experiments have compared the differences in the *λ* values between the warming and cooling phases, leading to the conclusion that they do not thermoregulate during the short term (Neill et al., 1976; Don and Neill William, 1979). These studies have mostly focused on the range of the thermal tolerance of SKJ; however, SKJ often dive beyond the thermocline and reach environments outside the critical temperature limits in the wild (Kiyofuji et al., 2019). In addition to physiological thermoregulation, further exploration of the behavioral aspect is required, as the free-ranging environment is completely different from controlled experiments (Brill, 1994). While laboratory studies have established a solid understanding of thermoregulation in SKJ, relatively few studies have comprehensively compared the thermoregulation of SKJ in terms of both behavioral and physiological aspects across their broad thermal range in the wild.

Physio-logging in free-ranging animals is a promising tool for accumulating long-term data, leading to a better understanding of their thermal physiological traits and responses to the natural environment (Fahlman et al., 2021). Accumulation of SKJ biologging data, including records of internal body temperature from previous studies (Aoki et al., 2017; Kiyofuji et al., 2019), allows us to extract new knowledge of their thermal physiology. Moreover, the distinct thermal habitats they encounter during migration (Aoki et al., 2017) enable us to identify specific thermal physiological traits for each thermal habitat. Building on previous tagging and recapture studies by our group, our study focuses on how the thermal physiology of SKJ explains the differences in its vertical behavior, particularly surfacing and diving, across various thermal environments during their northward migration. First, we classified thermal habitats using temperature and swimming depth records in a cluster analysis with newly added tagging data from a previous study (Aoki et al., 2017). Subsequently, we quantified the surfacing and diving behaviors in each thermal habitat, estimated the *λ* in the cooling and warming phases, and compared the values among thermal habitats to assess whether short-term thermoregulation occurred. Finally, we discussed how SKJ behavior addresses the spatial gap between migration ecology and fisheries in subtropical areas from the viewpoint of thermal physiology.

## 2 Materials and methods

### 2.1 Tagging procedure

Our tagging research is a part of a comprehensive tagging project described in previous studies (Aoki et al., 2017; Kiyofuji et al., 2019) and briefly mentioned herein. SKJ were captured by pole-and-line vessels at subtropical areas (20–24 N, 136–141°E) and off the Boso areas (30–40°N, 141–145 E) during February to March 2012–2015 and May to July 2013–2015, respectively. SKJ individuals with a fork length (FL) of 38–49 cm were selected for investigating the north migration process and were tagged on board with archival tags surgically inserted into the peritoneal cavity. Overall, 1251 tagged individuals were released. The electric tag type, LAT2910 (Length, 26 mm; diameter, 7.8 mm; Lotek Wireless Inc., Newfoundland, Canada), was used in this study, as it has previously been successful in recording behavioral data for SKJ with minimal effects from tag attachments (Kiyofuji et al., 2019). The stalk of the thin, flexible sensor records light intensity and external temperature. The main body, embedded in the peritoneal cavity, also has a sensor for recording internal temperature and pressure. These data are recorded at 30-s intervals, and the tag can continue recording for at least 1 year using an embedded battery. To date, 76 individuals were recaptured, and data were retrieved from 38 of them (Table 1). The others were not used for analysis because of technical issues (e.g., short recapture within 1 day of release or tag deterioration). Daily geolocation estimates based on light intensity for all the recovered tags have been already published in the study by Kiyofuji et al. (2019), and we referred to the literature for the corresponding geolocations.

**TABLE 1:** Recaptured tags used for the analysis.

Tag ID	Release				Recapture			
	FL cm	Date	Lat. (°N)	Lon. (°E)	FL (cm)	Date	Lat. (°N)	Lon. (°E)
411	42.5	08 February, 2012	23.40	129.67	Na	15 May 2012	30.41	130.24
476	42.0	15 February, 2012	20.40	136.20	44.0	21 May 2012	35.00	140.23
532	43.0	08 February, 2012	26.65	140.92	46.0	06 April, 2012	24.00	140.00
602	45.0	08 February, 2012	26.65	140.93	46.0	07 May 2012	31.55	139.83
1222	40.0	09 February, 2013	23.18	138.02	43.5	28 April, 2013	30.57	131.23
1316	40.0	16 March, 2013	20.40	136.18	45.0	31 May 2013	34.00	138.83
1328	39.0	10 March, 2013	24.85	141.13	46.0	07 June, 2013	33.33	139.67
1348	40.0	10 March, 2013	24.77	141.18	44.3	07 June, 2013	32.50	144.00
1359	38.0	10 March, 2013	24.85	141.13	44.3	08 June, 2013	33.38	139.22
1883	46.5	27 June, 2013	35.05	141.62	47.0	18 July, 2013	38.50	145.50
1886	48.0	17 June, 2013	34.93	143.93	51.5	07 August, 2013	37.00	147.00
1894	48.0	27 June, 2013	35.05	141.62	48.4	24 July, 2013	38.00	145.00
1897	48.0	01 July, 2013	35.52	145.35	48.0	14 July, 2013	38.72	147.75
1932	49.0	10 July, 2013	36.95	144.33	54.9	11 September, 2013	38.00	148.83
1950	46.0	11 July, 2013	36.18	142.15	47.0	27 August, 2013	40.00	145.00
1974	48.0	01 July, 2013	35.52	145.35	51.3	26 July, 2013	38.88	146.80
2410	47.0	14 March, 2014	20.43	136.02	47.1	6 April, 2014	20.33	136.08
2536	44.0	14 March, 2014	20.43	136.02	42.5	6 April, 2014	20.33	136.08
2601	45.0	28 May 2014	30.73	141.33	Na	01 August, 2014	37.33	147.00
2605	45.0	28 May 2014	30.73	141.33	48.0	25 June, 2014	36.00	148.50
2607	45.0	28 May 2014	30.73	141.33	48.0	16 June, 2014	34.67	144.50
2608	44.0	28 May 2014	30.73	141.33	45.0	21 June, 2014	38.30	141.50
2620	46.0	28 May 2014	30.73	141.33	46.4	24 June, 2014	35.50	147.50
2673	45.0	2 June, 2014	31.58	144.22	Na	25 June, 2014	35.67	147.83
2680	44.0	2 June, 2014	31.58	144.22	49.5	31 July, 2014	33.07	142.95
2746	44.0	2 June, 2014	31.63	143.98	44.2	27 June, 2014	35.03	150.05
2810	46.0	2 June, 2014	31.63	143.98	50.5	30 July, 2014	33.00	142.00
2822	45.0	11 June, 2014	34.33	148.40	Na	02 July, 2014	35.00	151.00
2832	43.0	2 June, 2014	31.59	144.22	46.0	20 July, 2014	34.65	145.87
3136	41.0	10 March, 2015	23.75	141.72	42.1	23 March, 2015	23.63	141.78
3164	39.0	10 March, 2015	23.75	141.72	42.0	20 April, 2015	22.00	140.00
3174	40.0	10 March, 2015	23.75	141.72	42.3	31 March, 2015	23.85	142.83
3230	38.0	12 March, 2015	23.48	141.95	Na	13 April, 2015	23.50	141.93
3417	41.0	10 March, 2015	23.75	141.72	Na	13 April, 2015	23.50	141.93
3690	43.0	21 May 2015	33.60	143.25	48.0	11 June, 2015	36.00	145.00
3735	46.0	2 June, 2015	33.12	149.05	48.0	11 July, 2015	37.00	145.00
3964	49.0	10 June, 2015	37.33	143.40	47.9	16 June, 2015	37.47	144.08
3969	48.0	10 June, 2015	37.33	143.40	52.4	21 June, 2015	38.00	143.52

### 2.2 Thermal habitat classification

**FIGURE 1:**
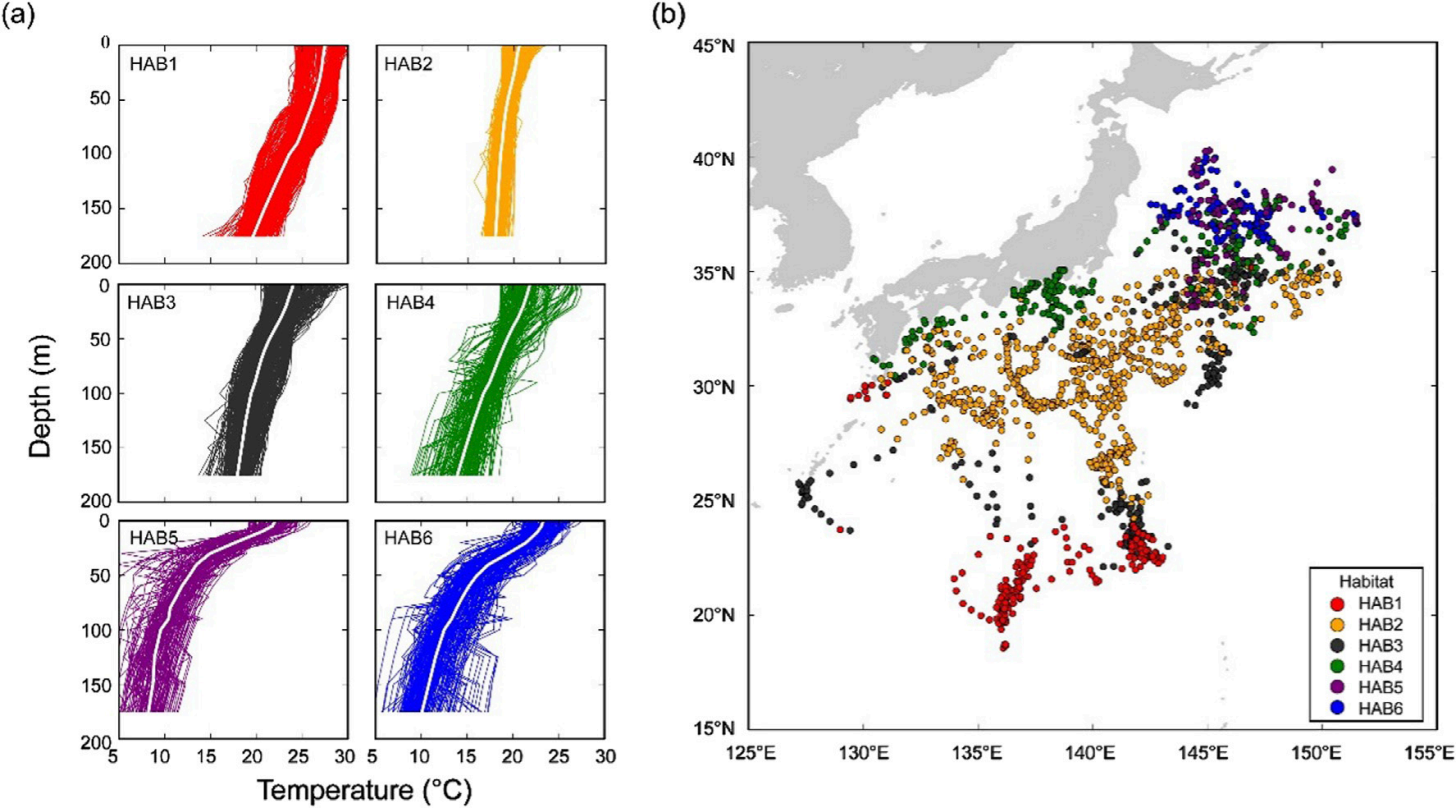
Thermal habitat (HAB) classification based on daily thermal profiles (0–200 m). **(A)** Vertical thermal profiles of the identified six habitats. The average temperature-at-depth of the profile is represented by a white line in each thermal habitat. **(B)** Daily geolocations of 38 individuals classified based on the thermal habitats. Each color represents habitat (HAB–-6) grouped by cluster analysis based on thermal profiles. Note that this figure was updated from the original figure in Aoki et al. (2017).

Spatial and temporal changes due to migration make the comparison of behavior and thermal physiologies in the same thermal habitat difficult. To address this, temperature profiles collected with electronic tags were used in a clustering analysis to characterize SKJ thermal habitats. The habitats utilized by SKJ during their northward migration were referenced from a previously reported study (Aoki et al., 2017), with some updates based on the addition of newly recovered tags. Thermal habitats were classified into six clusters using daily thermal profiles, calculated from pairs of swimming depth and ambient temperature records (Figure 1). The daily thermal profile was reproduced using the following procedures. The ambient temperature recorded each day was divided into the following bins based on the swimming depth: (i) 5-m depth (0–10 m), (ii) 10-m depth (10–100 m), and (iii) 25-m depth (100–200 m). As the surfacing behavior of SKJ made data collection at deeper depths difficult, the depth bins were set wider at greater depths. All temperature data within each depth bin was averaged as a representative temperature at the depth, and the average temperatures from 0 to 200 m were combined to reproduce the daily thermal profile. Missing values at each depth were replaced by the environmental data of World Ocean Atlas 13 (0.25° grid). Profiles in coastal areas for which there were no environmental data were removed due to the incomplete data set. Profiles collected on release and recapture days were also removed, as the data length for these days differed from other days at liberty. The complete set of daily thermal profiles was grouped using a hierarchical agglomerative clustering algorithm, which was implemented on a dissimilarity matrix calculated from Euclidean distances using Ward’s linkage method. This method was selected because it successfully produces compact groups in highly migrated species (Bestley et al., 2009; Aoki et al., 2017). In this study, we allocated the thermal habitats for the newly added data based on the six known thermal habitats (Aoki et al., 2017), indicating that our update focused on the reallocation of the thermal habitats to the newly added data.

### 2.3 Vertical behavior in each thermal habitat

A daily surfacing rate is a useful indicator for evaluating the encounter rate between fish and fisheries, particularly as SKJ are vulnerable to surface fishery. To understand the basic features of the surfacing rate in each thermal habitat, a daily surfacing rate during the daytime was examined by assessing the proportion of time spent at the surface (≤10 m). The effect of thermal habitats on the daily surfacing rate was examined via the generalized linear mixed effect model (GLMM) using a binomial distribution with a logit link function in the “glmer” function of the “lme4” package in R software (R Foundation, Vienna, Austria). The purpose of this analysis is to evaluate the effect of thermal habitat on the daily surfacing rate. We modeled the daily surfacing rate as a response variable and the thermal habitat effect (HAB) as a fixed effect of the explanatory variable. The probability (*π*) of SKG being distributed at depths shallower than 10 m was calculated from the number of data on the surface (Surface) and the number of data during the day (Total), expressed using the following Equation 1:

$${\text{Surface}}_{ij}=\text{Bin }\left(\pi_{ij,},{\text{Total}}_{\text{ij}}\right)$$ (1)

$$logit\left(\pi_{ij}\right)=\beta_{k}\times HAB_{k}+z_{i}+\varepsilon_{ij},z_{i}\;∼\;N\;\left(0,\sigma_{TAGID}^{2}\right)$$

where *Surface*_*ij*_ represents the number of data in the surface (≤10 m) during daytime for observation *j* in each fish *i* and *β*_*k*_ represents the effect of *HAB*_*k*_ (k = 1, 2, 3, … 6). As observations were repeated measures collected from the same individuals, we incorporated individual fish as random intercept effect (*z*_*i*_), assuming them to be normally distributed with mean 0 and variance *σ* ^*2*^_*TAGID*_. In the binomial distribution, the variance is automatically determined when the mean is determined, resulting in overdispersion, where the variance of the observed data is larger than the expected one. Therefore, we applied two models for the error structure: (1) a model that only considers the individual effect, and (2) an observation level model that includes both individual effect and the error *ε*_*ij*_ at each observation. The choice of the best model was determined based on the Akaike Information Criteria (AIC). In addition, we used the dispersion statistic to assess overdispersion by calculating the sum of squared Pearson residual divided by the number of observations, minus the number of parameters. We then confirmed that the overdispersion was less than 1 (Zuur et al., 2013). We finally selected the observation level model as it exhibited a lower AIC (18156) than the model that considers only individual effects (450111). Dispersion static (0.06) did not indicate overdispersion (Zuur et al., 2013).

### 2.4 Effect of diving behavior on body temperature

Thermal differences between body and water temperature during dives provide a simple way to assess the effect of diving behavior on body temperature (Kitagawa et al., 2007). We examined the thermal difference during dives by averaging the thermal difference and water temperature during dives. Each dive event was extracted from the recorded swimming depth data as follows: The beginning of the dive was defined as the last point where SKJ descended to the depth (>10 m), and the end of the dive was defined as the first point where SKJ ascended back to the depth (<10 m). Data from depths shallower than 100 m during the dive were not used in this analysis, as the purpose of this analysis was to examine the effects of temperature changes during the dive on body temperature, which requires data outside their typical vertical range (Average swimming depth is 99 m; Kiyofuji et al., 2019).

Dive duration is another important variable for surface fisheries because it provides an estimate of the time it takes for SKJ to reappear at the surface after a dive. The particular concern here is how the dive duration changes in association with the thermal gradient in each thermal habitat. Dive duration was calculated as the time elapsed from the beginning to the end of the dive, and the thermal gradient between 0–10 m and 90–100 m was used as an indicator of the thermocline. As the dive duration exhibits a non-linear relationship with the thermal gradient, we applied generalized additive mixed effects models (GAMMs) with normal distribution in the “mgcv” package of R software to evaluate the effect of thermal gradient (*Grad*) on dive durations (*Dive*). We incorporated the thermal gradient as an explanatory variable (fixed effect) and dive duration as the response variable, as presented in the Equation 2 below for each observation *j* in each fish *i*.

$${\text{Dive}}_{ij}\;∼\;N\left(\mu_{ij},\sigma^{2}\right)$$ (2)

$$\mu_{ij}=\beta_{1}+f\left({\text{Grad}}_{ij}\right)+a_{i},a_{i}\;∼\;N\left(0,\sigma_{\text{TAGID}}^{2}\;\right)$$

where *β*_*1*_ is the intercept, and *f* is the smoothing function of the thermal gradient. Individual effect *a*_*i*_ was incorporated as a random effect, assuming it to follow a normal distribution with mean 0 and variance σ^2^_TAGID_. To account for errors in different thermal habitats, the variance in the equation was set to (1) σ2 when constant, and to (2) σ^2^_k_ when the variance changed among thermal habitats (k = 1, 2, … 6). Using AIC and likelihood ratio tests (Table 2), we finally identified the model that accounts for errors in different thermal habitats.

**TABLE 2:** Comparisons between two models [one with constant variance *σ* ^*2*^ and the other with variable variance $\sigma_{k}^{2}$ , (k = 1, 2···6)].

Model variance	df	AIC	BIC	logLik	Likelihood ratio	*p*-value
Constant	5	86,915	86,950	−43453	—	714
Variable	10	85,458	85,527	−42719	1,468	<0.0001

### 2.5 Thermal physiology

The whole-body heat transfer coefficient (*λ*) during the cooling and warming phases associated with dives was compared to investigate whether SKJ can thermoregulate during their dive. The coefficient was estimated from the recorded body (*T*_*b*_) and water (*T*_*a*_) temperatures using the heat budget model as follows (Holland et al., 1992; Aoki et al., 2017; 2020):

$$\frac{dT_{b}}{\text{dt}}=\lambda \left(T_{a}-T_{b}\right)+{\dot{T}}_{m}$$ (3)

This model describes that *T*_*b*_ changes depend on *T*_*a*_, *λ*, and heat production due to metabolism (*Ṫ*_*m*_). The magnitude of *λ* and *Ṫ*_*m*_ were estimated from the *T*_*b*_ curve during the warming and cooling phases (Aoki et al., 2017) using the method of non-linear least squares optimization. These phases used in the regression were extracted by analyzing whether the differences in the temperature data (*T*_*b*__t+1_- *T*_*b*t_) were positive or negative. Consecutive positive and negative data points that last for more than 10 min were selected as warming and cooling curves, respectively. To remove noisy data, *T*_*b*_ changes <1.0°C were excluded from this analysis.

The purpose of this analysis was to evaluate the differences in estimated *λ* between two the warming and cooling phases, providing evidence for physiological thermoregulation (Holland et al., 1992; Aoki et al., 2020; Hino et al., 2021). As the *λ* is a positive value, a GLMM with log-normal distribution was applied as follows Equation 4:

$$\lambda_{ij}∼\;N\left(\log\left(\mu_{ij}\right),\sigma^{2}\right)$$ (4)

$$\log\left(\mu_{ij}\right)={\text{Phase}}_{ij}+a_{i},\;a_{i}\;∼\;N\;\left(0,\sigma_{TAGID}^{2}\right)$$

where *λ*_*ij*_ represents the *λ* of individual *i* at observation *j*. Two phases of warming and cooling were incorporated as the fixed effect of categorical value (*Phase*_*ij*_), and the individual effect was expressed as *a*_*i*_, assuming it to follow the normal distribution with mean 0 and variance σ^2^_TAGID_. AIC in models with/without the *Phase*_*ij*_ was compared to evaluate the effect of the two phases in each HAB.

Estimated *λ* and *Ṫ*_*m*_ in each HAB were used to simulate dive durations required for *T*_*b*_ to reach the critical temperature of 18°C (Barkley et al., 1978; Kiyofuji et al., 2019), when SKJ descend from the surface to a depth of 200 m in each HAB. In this simulation, the following equation, based on Equation 3, was used.

$$\frac{T_{b}\left(t+∆t\right)-T_{b}\left(t\right)}{∆t}=\lambda \left(T_{a}\;\left(t\right)-T_{b}\left(t\right)\right)+{\dot{T}}_{m}$$ (5)

The median value of *λ* and *Ṫ*_*m*_ in the cooling phase, *T*_*a*_ at 200 m, and *T*_*b*_ in each HAB (Table 3) were incorporated into Equation 5. As the *λ* and *Ṫ*_*m*_ can vary depending on the specific temperature range reached during the cooling phase, we adopted the median λ values to estimate the most frequently utilized thermal ranges for each thermal habitat as a representative value of these parameters. *T*_*b*_ was simulated in every interval (Δ*t*) until 200 min. The simulated dive durations were compared with observed dive durations calculated in Section 2.4. (Effect of diving behavior on the body temperature). Note that while the simulated durations considered only the cooling phase, the actual dive durations included both the cooling and warming phases associated with dives.

**TABLE 3:** Parameters used for the dive simulation.

	HAB1	HAB2	HAB3	HAB4	HAB5	HAB6
Body temp. (°C)	24.51	20.58	22.18	22.14	24.2	23.49
Whole-body heat transfer coefficient *λ* (10^−3^s^-1^)	0.52	0.62	0.57	0.40	0.31	0.48
Metabolic heat production *Ṫ*_*m*_ (10^−3^°C·s^-1^)	1.39	0.70	1.00	0.77	1.29	1.40
Temp. 200 m (°C)	19.21	18.16	18.36	13.75	9.28	6.52
Observed duration (min)	47.0	61.1	45.2	34.2	15.1	5.2
Estimated duration (min)	—	—	—	42.6	46.2	17.2

## 3 Results

### 3.1 Thermal habitat classification

The daily geolocations of classified thermal habitats (*N* = 1385), based on the thermal profile with the updated data, covered the area of the Pacific region around Japan and captured the ocean currents (Figure 1). Overall geographical trends for the updated thermal habitats did not differ from those reported in the study by Aoki et al. (2017). However, our study provided more data for the area around the Boso region (30–35°N, 140–145°E), where spatial coverage was low in the original study, thereby strengthening the robustness of the classification. Looking more specifically at each thermal habitat, HAB1 (*N* = 213), mainly distributed south of latitude 25°N, had a warm surface temperature (SST: 25.0°C ± 0.8°C) with an isothermal layer extending from 0 to 50 m. Below a depth of 50 m, the temperature gradually decreased with increasing depth. HAB2 (*N* = 598) was widely spread out from 25°N to 30°N and had the lowest SST (20.5°C ± 1.0°C), with a mixed layer extending from 0 to 150 m. HAB3 (*N* = 244) was mostly found around the edge of HAB2, and its thermal profile exhibited a gradual decline from 0 to 150 m. Habitat 4 (*N* = 181) was distributed in coastal areas of the main island, Japan, and appeared close to the areas around HAB5 and 6. Further, the average temperature within depths ranging from 175 to 200 m was 13.8°C, which closely resembled the features of the Kuroshio, where the temperature at a depth of 200 m is 15°C (Stommel and Yoshida, 1972). HAB5 (*N* = 80) and 6 (*N* = 69) were found between 35°N and 40°N. Both habitats had strong thermocline, but the difference between the two thermal habitats was that the temperature at depths of 175–200 m in HAB6 was slightly colder than that in Habitat 5.

### 3.2 Characteristics of vertical distribution in each thermal habitat

**FIGURE 2:**
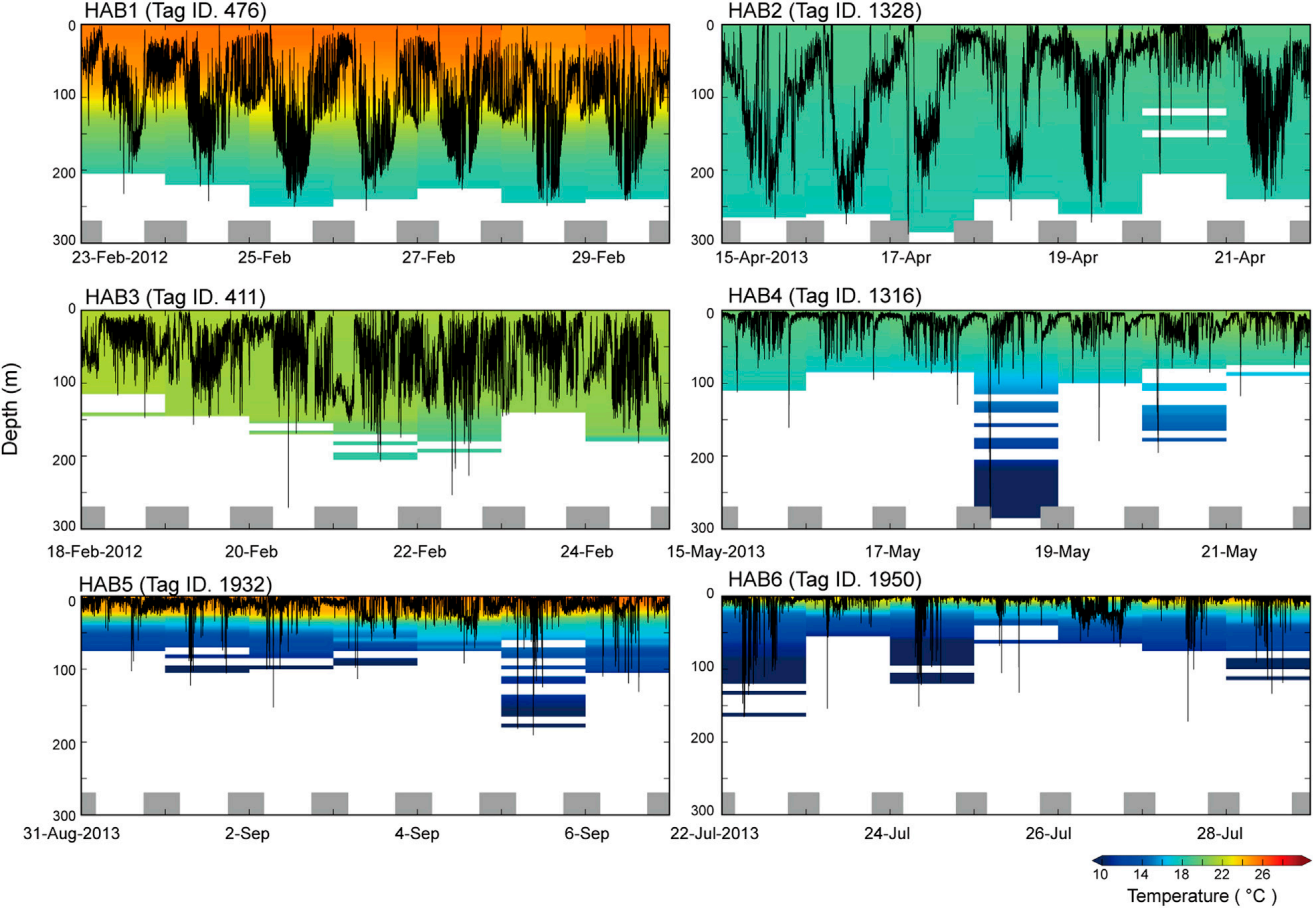
Examples (Tag ID 476, 1328, 411, 1316, 1932, 1950) of weekly time series showing vertical movements and daily ambient temperature profiles in thermal habitat (HAB) 1–6. The profiles were calculated from the time series for swimming depth and ambient temperature collected every 30 s. Bottom gray bars represent the night periods.

A weekly time series of vertical movements in each thermal habitat is presented in Figure 2 as a representative example. Tag ID 476 at HAB1 showed clear diurnal migration, with a daily average depth of 97–152 m deeper than that at night (50–96 m). Tag (1328 HAB2) also exhibited diurnal migration as in HAB1, and widely spread within the mixed layer (i.e., daily average depth: 17–131 m). The daily average depth of Tag ID 411 at HAB3 was 30–74 m, and the individual spent the daytime within the mixed layer. Tag (1316) at HAB4 showed a shallower distribution (daily average, 10–20 m) than the depths reached by HAB1–3, and the individual frequently dove around 79–179 m. It should be noted that the individual reached a depth of 286 m and experienced a water temperature of 8.3°C on 18 May 2013. Tag (1932 HAB5) exhibited a shallow daily average depth of 7–16 m, with a daily average water temperature of 24°C. Further, dive behavior beyond 100 m was frequently observed during daytime, and the individual experienced water temperatures as cool as 8°C. Tag (1950 HAB6) was distributed at a depth of 2–18 m on a daily average, and the individual experienced surface temperatures of 19°C–24°C.

**FIGURE 3:**
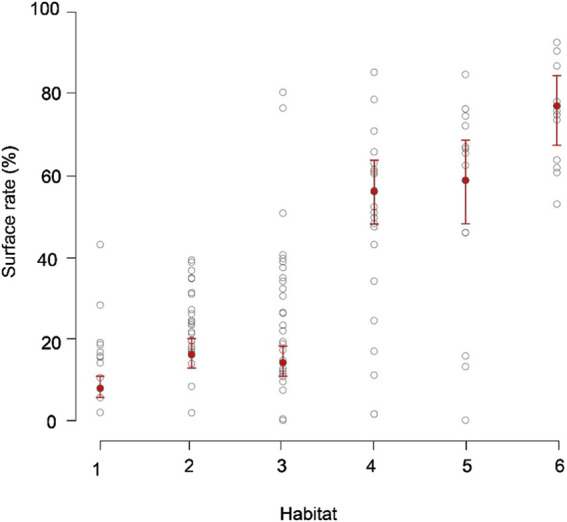
Probability of staying on the surface throughout the thermal habitats (HAB1–6). Circles show the average rate of staying on the sea surface (0–10 m) in the daytime of each individual and habitat. Note that daily surface rates were used in the statistical model, but the averages per individual and per thermal habitat are shown for visualization of the results. Red circles and bars indicate the estimated fixed effect and its 95% confidence intervals, respectively. The intervals were calculated by the fitted values plus or minus 1.96 × standard errors.

In total, SKJ distributions in HAB1–3 were mainly restricted to the thick mixed layer from 0 to 200 m (Figures 1, 2), and they did not experience drastic temperature changes during their dives. The temperature differences experienced during these dives were not large in HAB1–3. Contrastingly, SKJ in HAB4–6 spent most of their time on the surface, with occasional vertical dives to depths deeper than 100 m during the day. A remarkable change in ambient temperature was noted owing to strong stratification. These distribution trends are also reflected in the significant differences in the probability of being at the surface (>10 m) among thermal habitats (Figure 3; Table 4). Specifically, the lowest average probability was found in the southern area of HAB1 (20%), whereas the highest probability was noted in the northern area of HAB6 (90%).

**TABLE 4:** Result of estimated thermal habitat effect for surface model.

Habitat effect	Estimated *β*_*k*_	Std. error	z-value	Pr (>|z|)
HAB1	−2.442	0.174	−14.002	<2e-16
HAB2	0.808	0.167	4.837	1.32E-06
HAB3	0.594	0.174	3.423	<0.000619
HAB4	2.755	0.192	14.345	<2e-16
HAB5	2.861	0.252	11.353	<2e-16
HAB6	3.788	0.314	12.055	<2e-16

Z-values are obtained by dividing the estimated regression parameters using the standard errors, and the probability of absolute z-value is calculated by using the Z distribution.

### 3.3 Effect of diving behavior on body temperature

**FIGURE 4:**
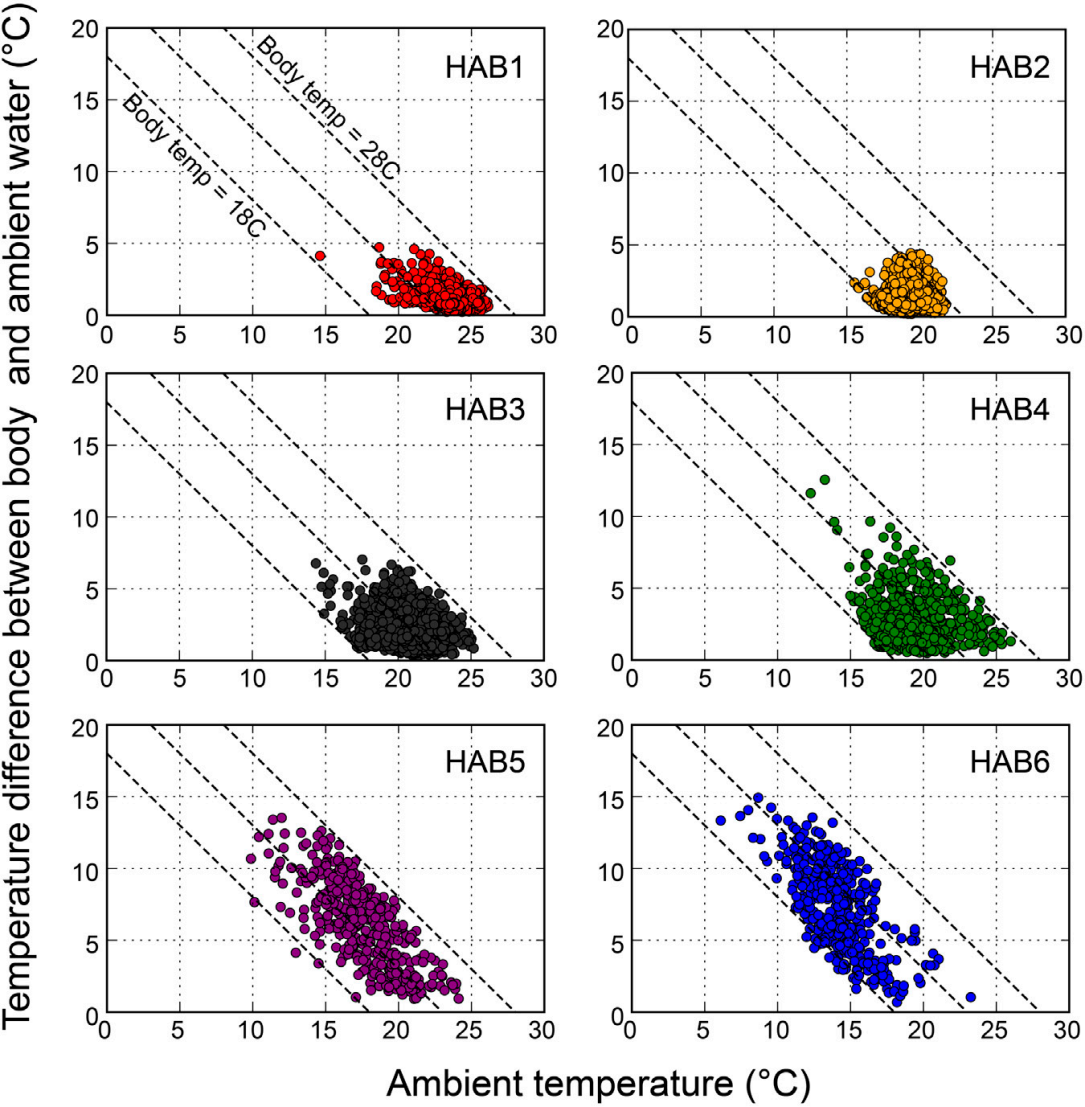
Relationships between the ambient temperature and the temperature difference between body (peritoneal cavity) and ambient during dives in habitats (HAB1–6), shown as average temperature values during one dive. Three dashed lines represent body temperatures of 18, 23, and 28°C, respectively.

The average water temperature and the thermal difference between body and water temperature during dives are presented in Figure 4. The overall body temperature ranged from 17.1°C to 28°C, and the thermal differences were more pronounced in the northern areas of HAB4–6 (0°C–15°C) compared to those in the southern areas of HAB1–3 (0°C–7°C). The minimum temperature during the dive was 6.2°C in HAB6.

**FIGURE 5:**
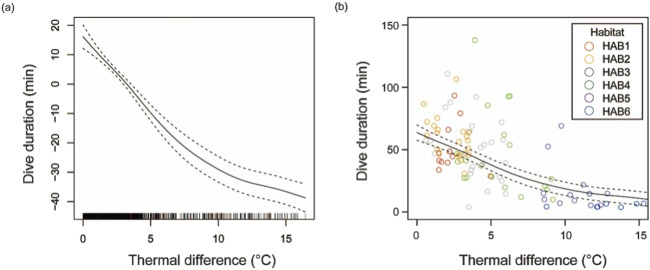
Dive durations of skipjack tuna from the surface to a depth beyond 100 m depth according to the strength of the thermal gradient (temperature difference between 0–10 m and 90–100 m layers). **(A)** Smooth function and **(B)** Dive duration (min) vs. thermal difference (°C) estimated using the generalized additive mixed modeling (GAMM). Solid and dashed lines show the fixed effect of the thermal gradient and its 95% confidence interval.

The dive duration significantly shortened with a strong thermal gradient (Figure 5; *f*(Difference_*ij*_) = 4.024, F value; 70.35, p < 0.001). The average dive durations in HABs with weak thermal gradients (HAB1–3) were 47.0, 61.1, and 45.2 min, respectively. In contrast, the durations in strong thermocline areas (HAB4–6) were 34.2, 15.1, and 5.2 min, respectively.

**FIGURE 6:**
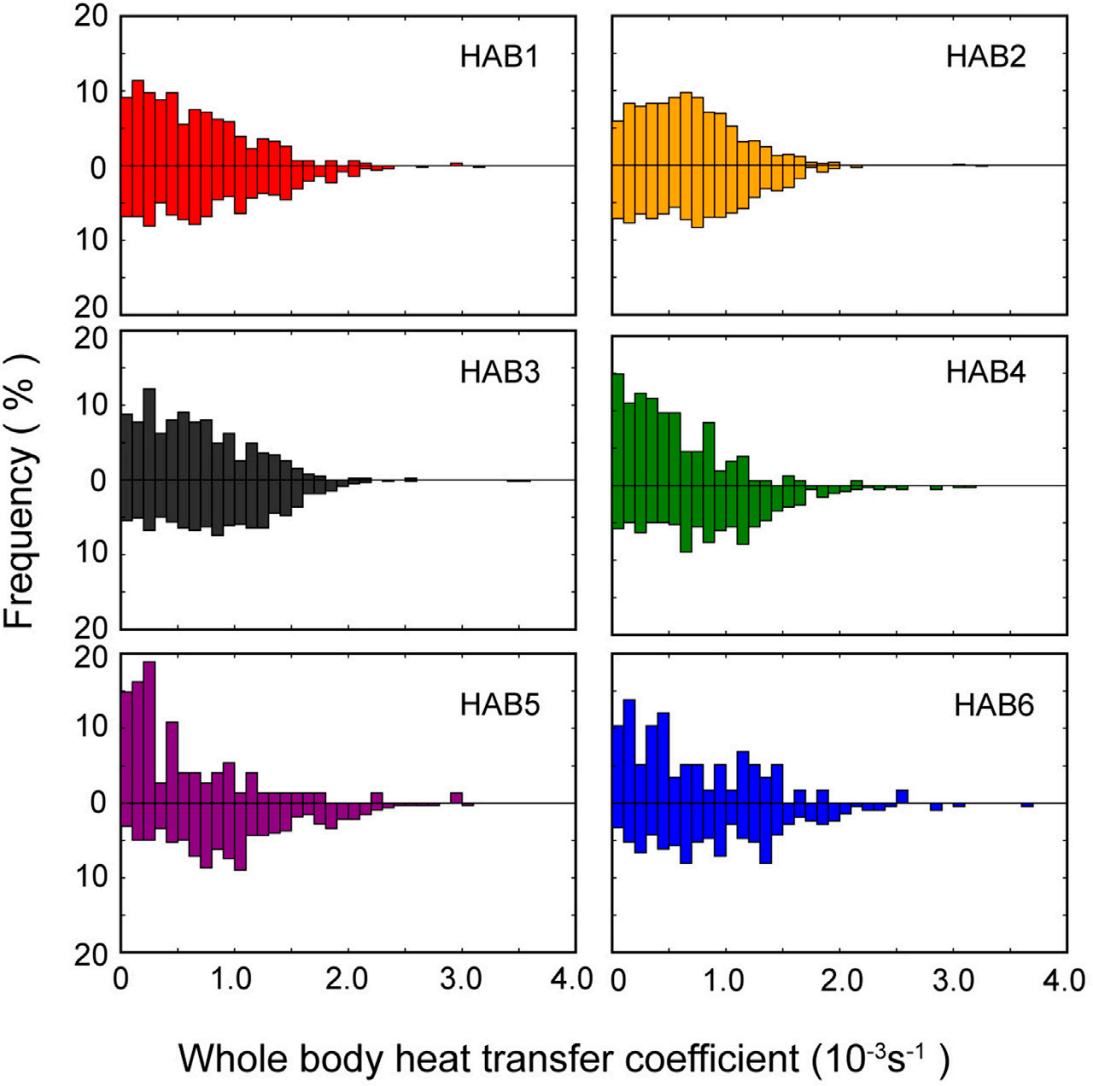
Frequency distribution of whole-body heat transfer coefficient in each thermal habitat. Upper and lower parts of each panel present the cooling (descending) and warming (ascending) phases, respectively.

The frequency distribution of the estimated median value of *λ* during the descent and ascent phases in each thermal habitat and the AIC values for models with phase effect (M1) and without the effect (M2) are presented in Figure 6 and Table 5, respectively. AIC for M1 showed lower values than those for M2, except in HAB2, and the phase effect was significant (*t*-static, p *<* 0.01). The estimated median *λ* in the descent phase was higher than that in the ascent phase (Table 5). The *λ* difference between the phases in HAB1 and 3 are 1.38 and 1.47, respectively, whereas the difference in HAB4–6 was 1.9–2.9. Focusing solely on the difference in *λ* among HABs, there was a tendency for a slight decrease in *λ* along with movements from low-latitude areas (HAB 1) to mid-latitude areas (HAB5–6). Calculating the reciprocal of the heat time constant (1/*λ*) provided a more intuitive understanding of the efficacy of their thermal physiology. This constant quantifies the degree of responsiveness to temperature changes and denotes the time required for the body temperature to change to 1/e (approximately 63.2%) of a specific temperature. The thermal constant time in HABs, calculated from *λ*, was 32, 27, 29, 42, 54, and 35 min for HAB1–6, respectively.

**TABLE 5:** Median values of whole-body heat transfer coefficient *λ* (10^−3^s^-1^) in the warming and cooling phases among thermal habitats and the comparisons between two models.

Habitat	Cooling		Warming		AIC	
	Number	*λ* (10^−3^s^-1^)	Number	*λ* (10^−3^s^-1^)	M1	M2
HAB1	306	0.52	478	0.72	2206	2221
HAB2	757	0.62	671	0.73	3799	3748
HAB3	384	0.57	600	0.84	2670	2697
HAB4	154	0.40	378	0.84	1481	1532
HAB5	74	0.31	322	0.91	1047	1099
HAB6	58	0.48	208	0.92	714	727

**FIGURE 7:**
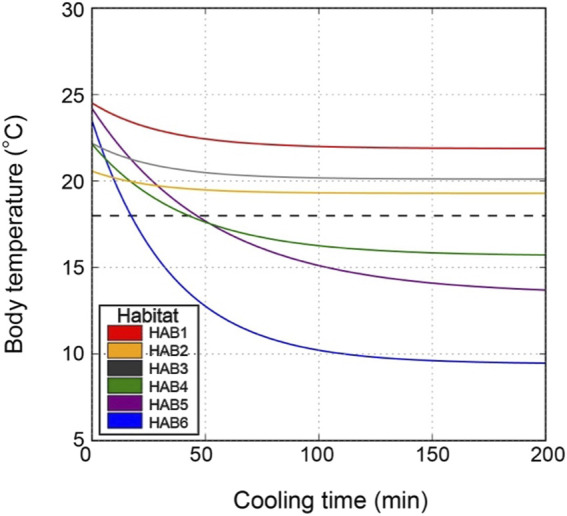
Simulation of body temperature change at a dive depth of 200 m in each thermal habitat (HAB). Initial body temperature, water temperature at a depth of 200 m, whole-body heat transfer, and metabolic heat production used in this simulation are presented in Table 3. Dashed horizontal line shows a critical temperature limit of 18°C estimated based on the study by Barkley et al. (1978).

In addition to this intuitive interpretation, we performed a more practical simulation for body cooling time specific to each thermal habitat. We calculated the time required for the body temperature to be cooled to 18°C when an SKJ individual dives to a depth of 200 m. The simulated dive time in HAB4–6 was 4, 46, and 17, respectively, which was longer than the actual observed dive durations (Figure 7; Table 3). In HAB1–3, the body temperature was not cooled to a critical temperature limit of 18°C, as the water depth at 18°C was deeper than 200 m.

## 4 Discussion

In the present study, we applied a physio-logging technique to estimate the thermal physiology parameter of *λ* during the cooling and warming phases associated with diving, based on body temperature records. Our findings provide evidence of thermoregulation in wild SKJ, showing several patterns in the differences in *λ* values between phases, largely attributable to their surrounding thermal profiles. Our findings on the thermoregulation in SKJ are not surprising, as this species is known as regional endothermy fish, possessing anatomically unique rete mirabilia, a feature commonly found in other tuna species (Carey and Teal, 1966; Brill, 1994). However, the new finding of changes in *λ*, which has not been reported in previous laboratory experiments (Neill et al., 1976; Stevens and Neill, 1979), further expands the interpretation of their thermoregulation ability specific in free-ranging environments. Identifying thermal physiology in the wild also provides mechanistic insights into why their surfacing behaviors are unique in each thermal habitat, as well as a quantitative evaluation of how their dive duration is linked to their physiology.

In this section, we evaluated the validity of the classification of thermal habitats in our study and then discussed how thermal physiology differs among thermal habitats, focusing on the short-term thermoregulation, while comparing our results to previous findings from laboratory experiments (Neill et al., 1976; Stevens and Neill, 1979). We also subsequently discussed the links between area-specific thermal physiology and the vertical behavior of surfacing and diving in each thermal habitat. Finally, we described the implications for the spatial heterogeneity of SKJ fishery grounds in the northwestern Pacific Ocean.

### 4.1 Validity of the classification of thermal habitats

In the present study, habitat classification was based solely on recorded depth and water temperature from tagged SKJ to avoid arbitrary spatial and temporal boundaries dependent on position estimates (Bestley et al., 2009; Aoki et al., 2017; Matsubara et al., 2024). This objective approach effectively captures the basin-scale oceanographic structures as a large water mass. Specifically, the thermal profile in HAB1 has a similar feature of the subtropical front around the latitude of 25°N in the western North Pacific (Kobashi et al., 2006), and the strong mixed layer in HAB2 corresponds to that of the subtropical mode water (Suga and Hanawa, 1995; Oka et al., 2023). Moreover, the thermal profile in HAB4 exhibited features similar to those of the Kuroshio, where the temperature at a depth of 200 m is 15°C, with broad overlap with the coastal regions of the Kuroshio current (Stommel and Yoshida, 1972). These consistencies with the description of the oceanic structure support the validity of our classification. However, there are limitations in identifying habitats as a single oceanographic area, leading to difficulty in interpreting the SKJ habitat-specific behavior, particularly in HAB3 and 4. Although the thermal profile is consistent within each HAB, the sparse locations of HAB3 (found north and south of HAB2) and HAB4 (found around HAB5 and 6) should be carefully considered when evaluating behavior in these thermal habitats. There is considerable variation in the surface rate in HAB3 and 4. However, given that these data points are relatively few, we assumed that their effects would be minimized when using average trends.

### 4.2 Characteristics of SKJ thermal physiology

The large thermal differences between body and water temperature during dives in low-latitude (HAB 1) areas compared to those in mid-latitude (HAB5–6) areas offer an intuitive understanding of the differences in their thermal physiology depending on the thermal habitats. High body temperature during dives can be explained by the change in the *λ* between the cooling and warming phases (Holland et al., 1992; Kitagawa et al., 2007; Aoki et al., 2020). Our observations of slight or little difference in *λ* in HAB1–3 are consistent with the results of Neill et al. (1976) and Stevens and Neill (1979) in SKJ (0.4–3.5 kg body weight) tested at water temperatures above 18°C, suggesting that this species was not physiologically able to regulate its body temperature. The minimal difference in *λ* observed in these areas is reasonable, as the water temperature at 200 m depth in these HABs was higher than the critical temperature limit of 18°C (Barkley et al., 1978). The agreement between the laboratory studies and our findings in the wild suggests that SKJ are not severely required to thermoregulate in the short term to maintain their body temperature within environments that fall within their thermal tolerance range.

In contrast, the *λ* in HAB4–6 differed by a factor of 2–3 between descent and ascent, indicating that SKJ physiologically regulated their body temperature during their diving behavior. Tropical tuna species, such as bigeye and yellowfin tuna, thermoregulate both behaviorally and physiologically by reducing *λ* during descent to prevent heat release from the body and increasing *λ* during ascent to absorb heat from the warmer water, allowing them and recover body temperature in a short period (Holland et al., 1992; Aoki et al., 2020). While the difference in *λ* for SKJ was not as pronounced as in bigeye tuna (with a 12-fold difference between phases), it was still slightly higher than that of yellowfin tuna (two-fold difference). This physiological thermoregulation in strong thermocline areas suggests that such mechanisms are common in tropical tuna and are vital for accessing depths below the thermocline that exceed their thermal tolerance in the wild.

The change in the *λ* between the cooling and warming phases is likely due to changes in blood flow during dives. Heat exchange in fish can be mainly divided into the effect of body inertia and internal body effects such as blood flow (Kitagawa and Kimura, 2006; Aoki et al., 2020). Although thermal inertia does not change in a short period such as diving behavior, changes in the blood flow can occur. According to a study that examined the relationship between blood circulation and respiration (Brill Richard and Bushnell Peter, 2001), the volume of blood output from the heart of SKJ remains constant, but changes in heart rate can alter the volume of blood output per unit time. As heat exchange occurs between arterial and venous blood in the counter-current heat exchanger developed in tuna (Carey et al., 1971), changes in heart rate are believed to alter heat exchange efficiency in the rete mirabile.

In addition to short-term thermoregulation, long-term thermo-conservation, driven by the body bulk effect, contributes to substantial thermal inertia, helping to maintain a high core temperature (Neill et al., 1976). In the Kuroshio–Oyashio transition area, only SKJ with an FL of >45 cm and a high-fat condition factor (≥20) are found in the waters north of the Kuroshio front. This suggests that large individuals meeting these criteria can move from relatively warm waters in the 20°C range to cold waters in the 18°C range (Nihira, 1996), a phenomenon called “size-screening”. However, the consecutive measurements of the daily average temperature during the northward migration revealed that SKJ (<45 cm FL) already experienced the lowest temperature of 18°C in areas around HAB2, which is further south of the Kuroshio–Oyashio transition area (Kiyofuji et al., 2019). The direct link between experienced temperature and thermal inertia, previously hypothesized as a size-screening phenomenon, has not been validated in individuals with archival tags in the Kuroshio–Oyashio transition area. However, it is noteworthy that slight decreases in *λ* during the cooling phase were observed alongside movements from low-latitude areas (HAB 1) to mid-latitude areas (HAB5 and 6). This observation could be interpreted as developments in thermal inertia associated with growth. In HAB5 and 6, the presence of the Oyashio current beneath the upper warm layer offers abundant food resources derived from high productivity (Yasuda, 2003; Sassa et al., 2007). Feeding while reducing the *λ* in these rich food areas would further contribute to lowering *λ* by increasing thermal inertia due to additional fat reserves (Aoki et al., 2017). This process could initiate a positive feedback loop, stimulating enhanced exploitation of food resources within this area (Nihira, 1996). The body bulk effect is commonly developed in marine animals as they grow (Nakamura et al., 2020); however, the developmental shift to regional endothermy, associated with unique anatomical features, occurs more rapidly in juvenile tuna (Kitagawa and Fujioka, 2017; Hino et al., 2021; Kitagawa et al., 2022). Although increases in thermal inertia in SKJ were subtle in our study, further investigation targeting individuals with a wide variety of body sizes and fat contents is necessary to capture the drastic shift in their thermos-conservation ability, particularly along the ontogenetic shift.

### 4.3 Links between thermal physiology and vertical behavior

Surface-oriented behavior and short dive durations in SKJ are likely behaviors to avoid exposure to cold temperatures (Kitagawa et al., 2007; Lawson et al., 2010; Matsumoto et al., 2013; Matsubara et al., 2024). As high energy demand species, tuna must meet high oxygen demands (Brill Richard and Bushnell Peter, 2001; Bernal et al., 2017; Humphries et al., 2024). Prolonged exposure to cold temperatures would reduce oxygen supply and cardiac output, resulting in an increased risk of hypoxia (Bushnell et al., 1990; Shiels et al., 2015). Although SKJ possess the physiological ability to thermoregulate in the short term to maintain stable body temperature, as mentioned above, this ability is not sustainable beyond a certain duration. Our comparison of the body cooling stimulation with actual dive duration provided field evidence of how dive duration in SKJ is determined based on thermal physiology. An observed dive duration shorter than the simulated cooling time indicates that SKJ returned to the surface before their body temperature decreased to the critical level of 18°C (Barkley et al., 1978), which can be interpreted as behavioral thermoregulation (Kitagawa et al., 2007). During northward migration, the shallower the depth at which the 18°C threshold is reached (Kiyofuji et al., 2019), the more frequent the surfacing behavior and the shorter the dive duration. Therefore, areas with shallow depths, where this lower thermal tolerance is present, such as the temperate areas, would result in the vertical distribution of SKJ being near the surface with occasional short dives. In contrast, the relatively few restrictions posed by low thermal tolerance in subtropical areas allow SKJ to spend most of the daytime throughout a wide range of water columns.

The diving behavior of tuna offers advantages in encountering food resources and is particularly essential in areas where surface prey is scarce (Kitagawa et al., 2004; Walli et al., 2009; Bauer et al., 2017). Successful foraging associated with diving is related to both access to prey depths and long dive duration, which maximize prey encounters (Josse et al., 1998; Kitagawa et al., 2007; Walli et al., 2009). Prolonging the dive duration in subtropical areas, which are poor prey environments for SKJ (Aoki et al., 2017), is reasonable as it enables them to forage for long periods across a wider range of the water column. Although SKJ in subtropical areas expend three times more energy on feeding than they do in temperate areas, their estimated food intake is still only half of what is observed in temperate areas (Aoki et al., 2017). In contrast, the strong thermocline in temperate areas restricts the diving period of SKJ (Kitagawa et al., 2007; Lawson et al., 2010), which increases the risk of unsuccessful foraging. However, the rich prey environment in temperate areas (Aoki et al., 2017; Fujioka et al., 2018; Muhling et al., 2022) allows them to meet their high energy demands, even within the short foraging period.

### 4.4 Implications for SKJ surface fishery

The concept of prey-predator encounter based on the overlap of depths and times within vertical habitats is extendable to fishing activities between prey (SKJ) and predator (human) (Post et al., 2008; Lennox et al., 2017). Given that SKJ is mainly exploited by the purse seine and pole-and-line fisheries, which rely on visually locating schools swimming at the surface (Gaertner et al., 1999; Matsubara et al., 2022), changes in the vertical behavior among habitats would explain the spatial heterogeneity of scarce catch being reported in subtropical areas. The comprehensive behavioral dataset obtained from subtropical and temperate areas in this study, combined with previous literature from tropical regions, provides a valuable opportunity to compare and evaluate changes in vertical behavior. Our findings of non-surfacing behavior and long dive duration in subtropical areas may serve as a plausible explanation for the decrease in the encounter rate by surface fisheries. Nevertheless, a slight catch can be observed in subtropical areas (Williams, 2023), probably due to the increased chances of encounter during the limited time of dawn and dusk periods when SKJ distribution shifts between day and night. Notably, the habitat in subtropical areas would not be optimal for surface fisheries in terms of efficiency when searching schools on the surface. On the contrary, the surface-oriented behavior and shorter dive durations observed in temperate areas would facilitate the discovery of schools and subsequent catches by fisheries (Matsubara et al., 2024). While surface rates for individuals in tropical areas are unavailable, research on SKJ (53–73 cm FL) in tropical areas, both associated and unassociated with floating aggregate devices (FADs), indicates shallower depths (10.4 and 44 m during the day, respectively) (Schaefer and Fuller, 2007) than those observed in subtropical areas (HAB1, 97–152 m) in our study. In addition, SKJ unassociated with FADs showed surface-oriented behavior (less than 10 m below the surface for periods >10 min), ranging from 5 to 23 events in a day, each lasting 10–214 min per event (Schaefer and Fuller, 2007). The primary drivers of the surface-oriented behavior and relatively shallow depths in tropical areas remain unclear. However, it is plausible that this behavior enhances encounter rates for surface fisheries in tropical areas compared to those in subtropical areas. Therefore, integrating our findings on the shift in vertical behavior driven by thermal physiology in subtropical and temperate areas with previous literature from tropical areas explains why SKJ fishery grounds predominantly thrive in tropical and temperate regions (HAB4–6) in the western Pacific Ocean, consequently resulting in a spatial gap in subtropical areas.

Finally, this study established a link between the vertical distribution of SKJ and their vulnerability to fisheries by adding a novel component: the response of this species to thermal environments in the wild. A better understanding of the physiological mechanisms controlling vertical habitat use in the water column would offer ecologists a more mechanistic understanding of depth-related habitat, helping them predict the hotspots associated with changes in thermal profiles driven by ocean environmental variations. The predicted decline in the thickness of STMW in HAB2 (Oka et al., 2023) and the shoaling of the mixed layer in the Kuroshio Extension area (Zhang et al., 2016) in HAB5 and 6 highlight the importance of exploring thermal physiological responses. This knowledge is essential for predicting the redistribution of hot and cool spots for SKJ fisheries under these future climate change scenarios (Sunday et al., 2012). Such evaluations would reduce the uncertainty in future stock management and bring us closer to achieving sustainable fisheries (Lehodey et al., 2013).

## 5 Summary

This study investigated the vertical behavior and thermal physiology of skipjack (SKJ) during their northward migration from subtropical to temperate areas in the western Pacific Ocean using a physio-logging technique. We quantified the surfacing rate on the surface and dive duration and estimated the whole-body heat transfer coefficient (*λ*) based on body temperature records as an index of their thermal physiology. SKJ exhibited non-surface-oriented behavior with extended dive duration in the southern mixed layer areas, whereas they demonstrated surface-restricted behavior with occasional short dives in northern areas with strong thermocline. Notably, SKJ in northern areas exhibited greater thermoregulation (with differences in *λ* of 2–3-fold between the warming and cooling phases associated with diving) than those in southern areas (minor differences in *λ* between the phases). This provides new field evidence of short-term thermoregulation during dives in SKJ, which exhibit distinct patterns across the thermal habitats. This adjustment in *λ* during the phases in the northern areas allows SKJ to minimize heat release from the body by reducing *λ* during the cooling phase in descent and to absorb heat from the warm water near the warm surface and quickly recover body temperature by increasing *λ* during warming phase in ascent. Non-surface-oriented behavior with a long dive duration in warm subtropical areas would enable SKJ to forage efficiently in poor prey environments without requiring thermoregulation. The surfacing behavior, with occasional short dives in temperate areas, can be interpreted as a consequence of thermoregulation, avoiding exposure to cold temperatures beyond the thermocline while foraging in rich prey environments. The spatial difference in thermal habitat suitability would explain why SKJ exploitation by surface fisheries does not thrive in subtropical areas from the viewpoint of encounter rate by fisheries, which rely on the overlapping depth and time at the surface. Our findings of SKJ thermal physiology and its implications for vertical behavior and fishery vulnerability underscore the importance of considering thermal physiology in predicting species responses to ocean environmental changes, thus providing more plausible future stock estimates.

## Data Availability

The raw data supporting the conclusions of this article will be made available by the authors, without undue reservation.
